# Kaposi’s Sarcoma-Associated Herpesvirus ORF21 Enhances the Phosphorylation of MEK and the Infectivity of Progeny Virus

**DOI:** 10.3390/ijms24021238

**Published:** 2023-01-08

**Authors:** Tatsuo Yamaguchi, Tadashi Watanabe, Yuki Iwaisako, Masahiro Fujimuro

**Affiliations:** 1 Department of Cell Biology, Kyoto Pharmaceutical University, Kyoto 607-8412, Japan; 2 Department of Virology, Graduate School of Medicine, University of the Ryukyus, Okinawa 903-0215, Japan

**Keywords:** Kaposi’s sarcoma-associated herpesvirus, MEK, thymidine kinases, lytic replication, infectivity, ORF21, herpesvirus

## Abstract

Kaposi’s sarcoma-associated herpesvirus (KSHV), also known as human herpesvirus-8, is the causative agent of Kaposi’s sarcoma, Castleman’s disease, and primary effusion lymphoma. Although the functions of the viral thymidine kinases (vTK) of herpes simplex virus-1/2 are well understood, that of KSHV ORF21 (an ortholog of vTK) is largely unknown. Here, we investigated the role of ORF21 in lytic replication and infection by generating two ORF21-mutated KSHV BAC clones: ORF21-kinase activity deficient KSHV (21KD) and stop codon-induced ORF21-deleted KSHV (21del). The results showed that both ORF21 mutations did not affect viral genome replication, lytic gene transcription, or the production of viral genome-encapsidated particles. The ORF21 molecule-dependent function, other than the kinase function of ORF21, was involved in the infectivity of the progeny virus. ORF21 was expressed 36 h after the induction of lytic replication, and endogenously expressed ORF21 was localized in the whole cytoplasm. Moreover, ORF21 upregulated the MEK phosphorylation and anchorage-independent cell growth. The inhibition of MEK signaling by U0126 in recipient target cells suppressed the number of progeny virus-infected cells. These suggest that ORF21 transmitted as a tegument protein in the progeny virus enhances the new infection through MEK up-regulation in the recipient cell. Our findings indicate that ORF21 plays key roles in the infection of KSHV through the manipulation of the cellular function.

## 1. Introduction

Kaposi’s sarcoma-associated herpesvirus, known as human herpesvirus-8 (HHV-8), is a member of the gammaherpesvirus subfamily [1]. Human herpesviruses are classified into three subfamilies: *alphaherpesvirinae* (herpes simplex virus-1 (HSV-1), herpes simplex virus-2 (HSV-2), and varicella zoster virus (VZV)), *betaherpesvirinae* (human cytomegalovirus (HCMV), human herpesvirus-6A (HHV-6A), human herpesvirus-6B (HHV-6B), and human herpesvirus-7 (HHV-7)), and *gammaherpesvirinae* (Epstein–Barr virus (EBV) and KSHV). These human herpesviruses have a highly conserved replication system; however, their pathological features are divergent. KSHV is closely associated with Kaposi’s sarcoma, primary effusion lymphoma (PEL), and multicentric Castleman’s disease [2,3,4]. KSHV establishes a life-long infection in human B-cells or vascular endothelial cells and exists in either a latent or lytic state. During latent infection, the KSHV genome circularizes to form an episome in the nucleus of the infected cell and expresses several latent-associated gene products and microRNAs, which contribute to the promotion of cell proliferation and anti-apoptosis activity. KSHV shifts its life cycle from a latent to a lytic infection by the expression of a replication and transcription activator (RTA/ORF50). During lytic replication, lytic-related genes are translated, and virions are assembled and egress from the infected cell. As with other herpesviruses, KSHV virions consist of a linear, double-stranded DNA genome enclosed within an icosahedral capsid shell, tegument proteins, and a viral envelope composed of a single lipid bilayer and envelope glycoproteins [5].

The tegument protein KSHV ORF21 is expressed as a late gene product in lytic replication [6,7,8,9]. Because of its homology with the viral thymidine kinase (UL23) of alphaherpesviral HSV-1, KSHV ORF21 was originally reported as a homolog of HSV-1 UL23, viral thymidine kinase (TK) [10]. However, the amino acid sequence homology between KSHV ORF21 and HSV-1 UL23 is low (12.0%) [11]. The HSV-1 TK gene UL23 is conserved among the human herpesvirus, including the gammaherpesvirus subfamily [10]. The amino acid sequence homology with KSHV ORF21 from other gammaherpesviral homologs such as EBV BXLF1, equine herpesvirus 2 (EHV2) ORF21, and herpes virus saimiri (HVS) ORF21 is 28%, 31%, and 32%, respectively [10]. Human TK is an important enzyme that functions in the salvage pathway in the DNA synthesis of normal cells, and viral TKs of the alphaherpesvirus subfamily are well known to play an important role in viral genome replication. Therefore, nucleic acid analogs such as acyclovir are good therapeutic agents for alphaherpesviruses (i.e., HSV-1, HSV-2, and VZV). However, a comparative analysis of the capacities of HSV-1 TK and KSHV TK to mono-phosphorylate thymidine showed that the K_m_ of KSHV ORF21 was 60 times higher than that of HSV-1 UL23, and the V_max_ of ORF21 was 340 times lower than that of UL23 [12]. This means that in contrast with HSV-1 TK (UL23), KSHV TK (ORF21) has a very low ability to mono-phosphorylate thymidine. In addition, the KSHV gene encodes a thymidylate synthetase (ORF70), which can generate thymidine 5’-monophosphate without the reaction of mono-phosphorylation to thymidine [13].

KSHV ORF21 contains a conserved C-terminal kinase domain and a N-terminal proline-rich motif (PRM) [14]. PRMs are often found in the SH2 or SH3 domain-containing adapter proteins involved in the cell signaling molecules in the cytoskeletal remodeling [15,16]. In fact, ORF21 was found to disrupt adhesion plaques as a tyrosine protein kinase [14]. ORF21 harbors three autophosphorylation sites (Y65, Y85, and Y120) within the SH2 domain in the N-terminal region, and these Tyr residues are necessary for the interaction of ORF21 with Crk II (CT10 regulator of kinase II) or PI3-Kinase [17]. The C-terminal kinase domain of ORF21 was found to activate RhoA signaling, which is involved in cell contraction and blebbing [17]. Moreover, clinically approved tyrosine kinase inhibitors were found to inhibit the kinase activity of ORF21 [18]. Beauclair et al. constructed an ORF21-kinase activity deficient KSHV, in which three functionally important Gly residues (G260, G263, and G265) within the ATP binding pocket of ORF21 were substituted to three Val residues. The resulting ORF21-kinase activity deficient KSHV, named “kinase dead”, was analyzed, and it was clarified that the kinase activity of ORF21 and ORF21 autophosphorylation were not required for viral replication in KSHV-infected cells [18]. Meanwhile, ORF21 was reported to suppress Toll-like receptor (TLR)-2, TLR-4, and NF-κB signaling [19]. These findings suggest that ORF21 as a tyrosine kinase has beneficial effects for KSHV (e.g., immune response suppression, cell contraction, and cell migration).

Here, to disclose the role of ORF21 in KSHV replication and infection, we used a KSHV bacterial artificial chromosome clone (BAC16) and generated not only ORF21-kinase dead (kinase activity deficient) KSHV, but also ORF21-deleted (knockout) KSHV, in which stop codons were inserted into the ORF21 coding region. We compared the importance of the kinase activity of ORF21 and the ORF21 molecule during viral replication and evaluated the physiological role of KSHV ORF21 in host cells. The data showed that the ORF21 molecule was associated with de novo infection and upregulation of the cellular MEK signaling pathway.

## 2. Results

### 2.1. Construction of ORF21-Kinase Dead KSHV and ORF21-Deleted KSHV

**Figure 1:**
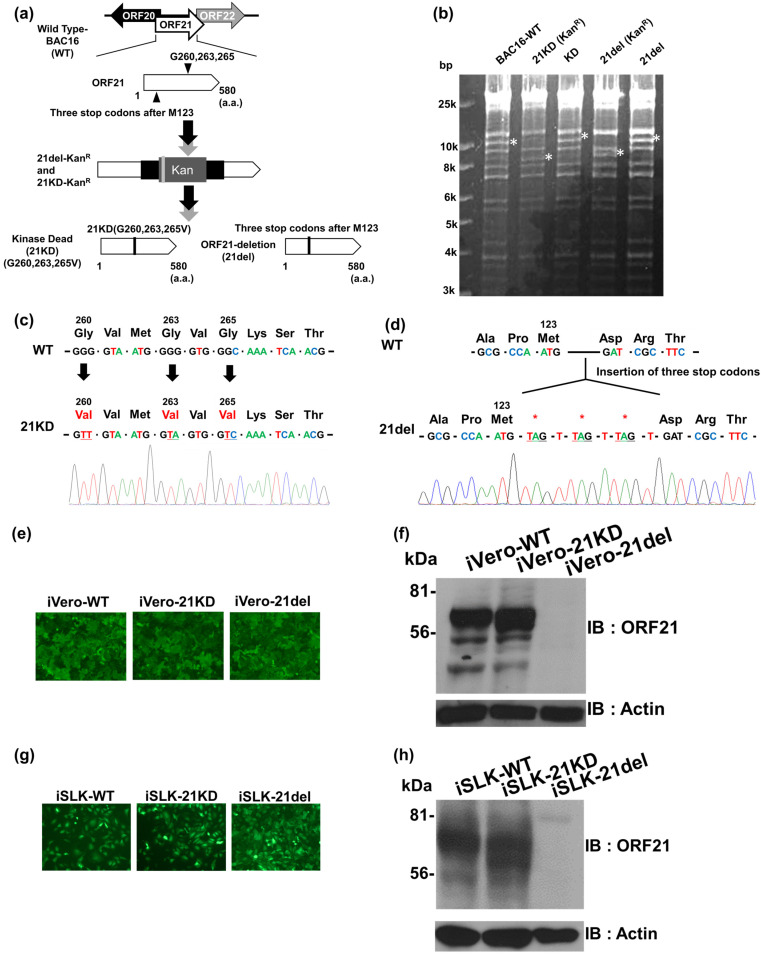
Construction of ORF21-kinase dead (21KD) and ORF21-deleted (21del) KSHV BAC16. (**a**) Schematic diagrams of the KSHV genome, including the ORF21-coding region. Two types of mutations (i.e., ORF21-kinase dead and ORF21-deleted) were generated in the ORF21 coding region (nucleotides (nt)35202 to nt36943) of the wildtype (WT) KSHV BAC16 clone (WT-BAC16) (GenBank accession number: GQ994935) by a two-step red recombination method. ORF21-kinase dead BAC16 (21KD-BAC16) had a kinase activity deficient mutation through the substitution of three Gly residues (G260, G263, and G265) within the ORF21 coding region in WT-BAC16. ORF21-deleted BAC16 (21del-BAC16) had a three stop codons insertion located after the fourth Met123-codon within the ORF21 coding region in WT-BAC16. a.a., amino acids. (**b**) Agarose gel electrophoresis of mutated BAC16 clones digested with Hind III. The asterisks indicate the insertion and deletion of the kanamycin resistance cassette in each BAC clone. The asterisks (*) indicate the insertion and deletion of a kanamycin-resistance cassette in each BAC clone. Original images are shown in Supplementary Figure S1a. (**c**,**d**) DNA sequencing results for ORF21 mutagenesis sites in 21KD-BAC16 and 21del-BAC16. (**e**,**g**) Establishment of mutated BAC16-harboring cell lines. WT-BAC16, 21KD-BAC16, and 21del-BAC16 were stably transfected into iVero cells (or iSLK cells), and the established BAC16-harboring cell lines were designated as iVero-WT (or iSLK-WT), iVero-21KD (or iSLK-21KD), and iVero-21del (or iSLK-21del), respectively. Each picture shows the fluorescence signal derived from the GFP gene in the BAC16 of the established cell lines. (**f**,**h**) Western blotting data showing the elimination of ORF21 expression in the lytic-induced iVero-21del and iSLK-21del cells. Cells were treated for 48 h with 1.5 mM NaB and 8 μg/mL Dox to induce lytic replication and were subjected to Western blotting using the anti-ORF21 polyclonal antibody. (**f**,**h**) Original images are shown in Supplementary Figure S1b,c.

To evaluate the importance of ORF21 in KSHV lytic replication, we generated ORF21-kinase dead (kinase activity deficient) KSHV and ORF21-deleted (knock out) KSHV, both derived from wildtype (WT) KSHV bacterial artificial chromosome clone 16 (BAC16) [20]. ORF21-kinase dead BAC16 (21KD-BAC16) was constructed by converting three Gly residues (i.e., Gly260, Gly263, and Gly265) to three Val residues in the ORF21 coding region of WT-BAC16 using a two-step markerless red recombination method (Figure 1a) [17,18]. ORF21-deleted BAC16 (21del-BAC16) was constructed by a three-stop codon insertion after the fourth Met123-codon (ATG) of the ORF21 coding region. Because the first, second, and third Met-codons of ORF21 overlapped with the neighboring ORF20 coding region, we targeted the fourth Met-codon (Figure 1a). The insertions and deletions of Kan resistance genes were analyzed by Hind III digestion (Figure 1b). Then, the replacement of Gly260, Gly263, and Gly265 by three Val residues in 21KD-BAC16 and the insertion of three stop codons into 21del-BAC16 were confirmed by Sanger sequencing (Figure 1c,d). To induce these recombinant KSHVs, doxycycline (Dox)-inducible RTA/ORF50-expressing SLK cells (iSLK) and Vero cells (iVero) were used as the virus-producing cells. To generate the recombinant KSHV-producing cell line, iSLK and iVero cells were transfected with each KSHV-BAC16 clone. WT-BAC16, 21KD-BAC16, and 21del-BAC16 were stably transfected into iVero cells (or iSLK cells), and the established BAC16-harboring cell lines were designated as iVero-WT (or iSLK-WT), iVero-21KD (or iSLK-21KD), and iVero-21del (or iSLK-21del), respectively. The BAC16-harboring cells could be easily distinguished through the detection of the fluorescence derived from the GFP gene in BAC16 (Figure 1e,g). In addition, the elimination of the ORF21 expression in lytic-replication-induced iVero-21del and iSLK-21del cells was validated by Western blotting using the anti-ORF21 rabbit polyclonal antibody (the right panels in Figure 1f,h).

### 2.2. ORF21 Is Localized in the Cytoplasm, and ORF21 and Its Kinase Activity Were Involved in Cell Contraction

**Figure 2:**
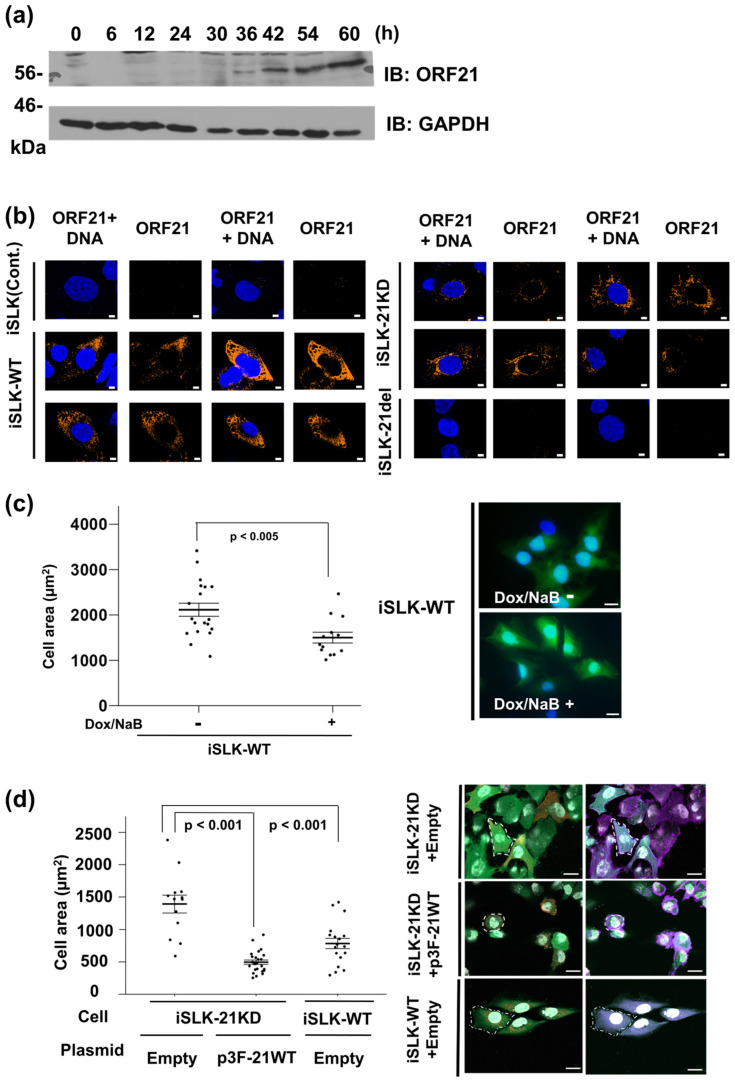
ORF21 is localized in the cytoplasm and is involved in cell contraction. (**a**) The endogenous expression of ORF21 protein in lytic-induced cells. iSLK-WT cells were treated for 0–60 h with NaB and Dox for lytic reactivation, and lytic-induced cells were analyzed by Western blotting using the anti-ORF21 polyclonal antibody. Original images of the blotting are shown in Supplementary Figure S2. (**b**) Localization of endogenously expressed ORF21 protein in lytic-induced cells. iSLK-WT cells were treated with 1.5 mM NaB and 8 μg/mL Dox for 48 h and analyzed with IFA. ORF21 protein (orange) and DNA (blue) were stained with the anti-ORF21 polyclonal antibody and DAPI, respectively. Scale bars represent 5 µm. (**c**) Effect of endogenous ORF21 expression on cell contraction in iSLK-WT during lytic replication. iSLK-WT cells were treated with (or without) Dox and NaB for 48 h and analyzed with a fluorescence microscope. Scale bars represent 20 µm. (**d**) Effect of exogenous ORF21 expression on cell contraction in iSLK-21KD cells during lytic replication. iSLK-21KD cells were transiently transfected with either 3×Flag-ORF21 wildtype (p3F-21WT) or an empty plasmid and cultured with Dox and NaB for 48 h. The left images show the GFP signal (green) derived from KSHV-BAC16, ORF21 (red), and nuclei (white). The right images show phalloidin signal (violet), GFP signal (green), and nuclei (white). The cell areas of the GFP- and phalloidin-positive cells were measured. Scale bars represent 20 µm. (**c**,**d**) *p* < 0.001 and *p* < 0.005 indicate a statistically significant difference.

The expression period of ORF21 mRNA has been estimated by microarray analysis and next-generation sequencing such as RNA-Seq [6,7,8], and the localization of ORF21 protein was reported to be in the cytoplasm by overexpression studies using the tagged ORF21-expression plasmid [17]. Therefore, we analyzed the expression timing and localization of the endogenously expressed ORF21 protein in lytic-induced cells using the anti-ORF21 rabbit polyclonal antibody. iSLK-WT cells were treated with Dox and NaB to induce lytic replication, and ORF21 protein in the cell extract was analyzed by Western blotting using the anti-ORF21 antibody. The results show that ORF21 was rapidly expressed between 30 and 36 h after the induction of lytic replication (Figure 2a). Next, iSLK without BAC16 (control), iSLK-WT, iSLK-21KD, and iSLK-21del cells were treated with Dox and NaB, and the fluorescence signal of the cells was analyzed using a fluorescence microscope. The endogenously expressed WT and ORF21 kinase dead proteins were distributed in the whole cytoplasm of the lytic-induced cells (Figure 2b). Furthermore, to gain information regarding ORF21 function within the host cell, we analyzed the effect of ORF21 expression on the cell contraction and morphology in KSHV-harboring cells in the lytic phase. iSLK-WT cells were treated with (or without) Dox and NaB, and the fluorescence signal of the cells was analyzed using a fluorescence microscope. The data showed that cell areas of lytic-induced iSLK-WT cells were decreased compared with those of non-induced iSLK-WT cells (Figure 2c). Next, iSLK-21KD cells were transiently transfected with Flag-tagged ORF21 and cultured with Dox and NaB. As the positive control, iSLK-WT cells transfected with empty plasmid were treated with Dox and NaB cells and were also analyzed. The cell areas of empty plasmid-transfected iSLK-21KD cells were increased compared with those of empty plasmid-transfected iSLK-WT cells. However, the cell areas of ORF21-transfected iSLK-21KD cells were decreased compared with the empty plasmid-transfected iSLK-21KD cells (Figure 2d). These suggest that ORF21, including the kinase activity of ORF21, induces cell contraction under KSHV lytic infection.

### 2.3. ORF21 Does Not Affect Intracellular Viral DNA Replication or Lytic Genes Transcription

**Figure 3:**
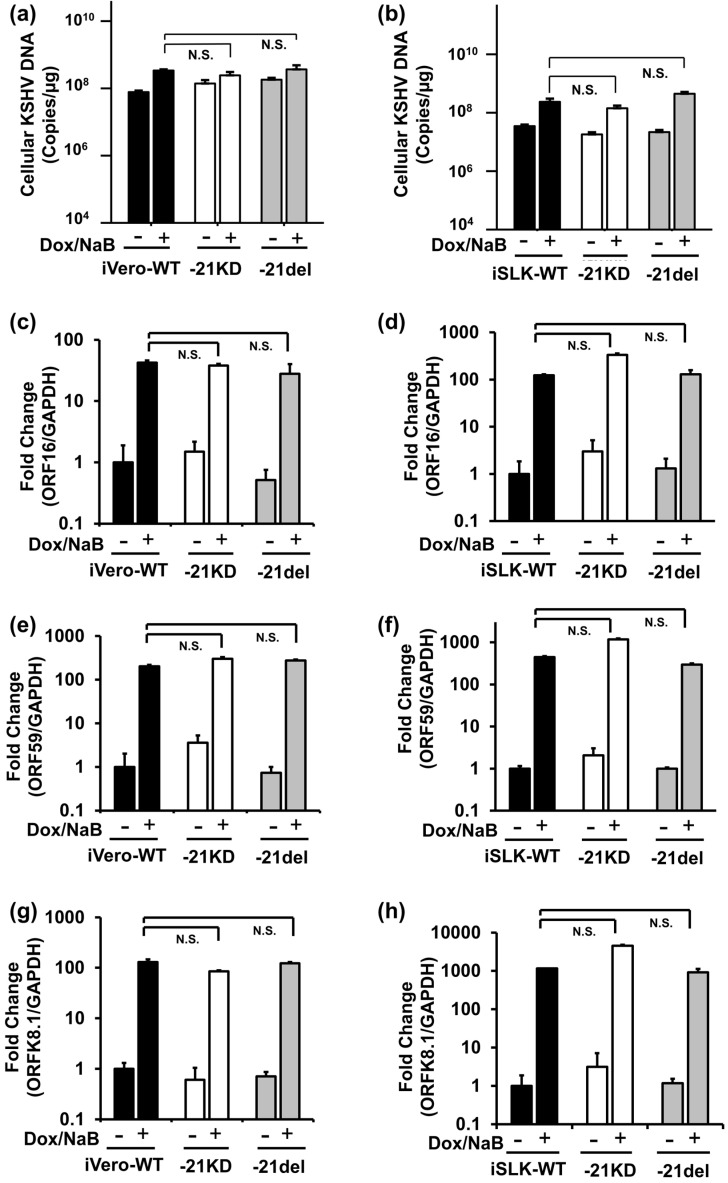
The effects of ORF21 and ORF21-kinase activity on the replication of intracellular viral DNA and the transcription of the lytic genes. Viral DNA replication (**a**,**b**) and viral gene transcription (**c**–**f**) in iVero (or iSLK) cells harboring WT-BAC16, 21KD-BAC16, and 21del-BAC16. The recombinant BAC16-transfected iVero cells (**a**,**c**,**e**,**g**) or iSLK cells (**b**,**d**,**f**,**h**) were treated for 48 h with Dox and NaB to induce lytic replication, and DNA genomes containing viral DNA were prepared from harvested cells. (**a**,**b**) The copies of intracellular viral DNA in the lytic-induced cells were measured using real-time PCR and normalized by the total amount of obtained DNA. (**c**–**h**) Quantities of mRNA expression levels of viral genes, immediate-early gene: ORF16 (vBcl-2); early gene: ORF59 (DNA processivity factor); late gene: K8.1 (glycoprotein) in the mutated KSHV-harboring cells. The total RNA was purified from lytic-induced cells and was subjected to RT real-time PCR. The values obtained from Dox- and NaB-untreated iSLK (or iVero)-WT cells were defined as 1.0. Statistical analysis was performed between Dox/NaB(+) groups in RT-qPCR. These results were not detected to have a statistically significant difference. (**a**–**h**) N.S., not significant.

To understand the virological importance of ORF21 in KSHV lytic replication, we examined the effects of ORF21 on viral genome replication and viral mRNA expression in lytic-induced cells harboring ORF21-mutated KSHV. iVero-WT, iVero-21KD, and iVero-21del cell lines were treated with Dox and NaB for the induction of lytic replication, and cells were harvested after 48 h. The intracellular viral genome from the harvested cells was quantified by qPCR. There was no change in the amount of viral DNA in iVero-21KD or iVero-21del cell line compared with those in iVero-WT cell lines (Figure 3a). The same experiment was repeated using iSLK cell lines, and similar results were obtained (Figure 3b). Next, to compare the lytic genes transcription between WT-KSHV- and ORF21-mutated KSHV-harboring iVero cells (Figure 3c,e,g) or iSLK cells (Figure 3d,f,h), the total RNA was prepared from the lytic-induced KSHV-harboring cells, and the mRNA expression of the lytic genes (immediate-early gene: ORF16, early gene: ORF59, and late gene: K8.1) was analyzed using RT-qPCR. However, we did not detect the effects of ORF21-kinase activity deficiency or ORF21-deletion on viral gene transcription in either cell line. These mean that ORF21 including its kinase activity is not involved in intracellular viral DNA replication or in lytic gene transcription in KSHV lytic replication.

### 2.4. ORF21 Has No Effect on the Production of Viral Genome-Encapsidated Particles

**Figure 4:**
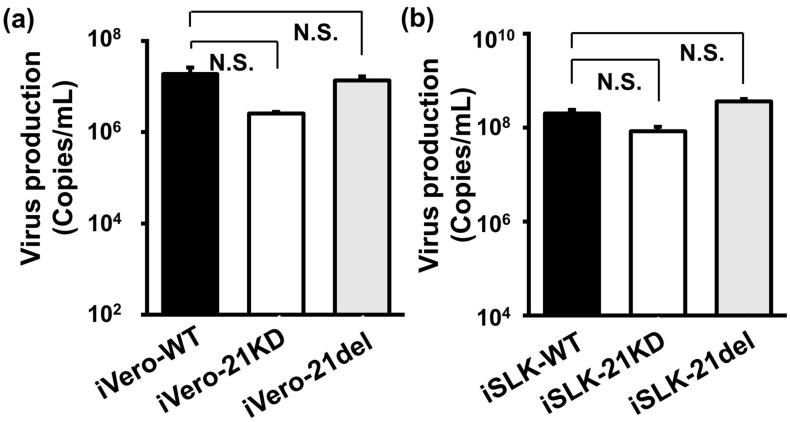
The effects of ORF21 on the production of cell-free genome-encapsidated particles. (**a**) Recombinant BAC16-harboring iVero cells (iVero-WT, iVero-21KD, and iVero-21del) or (**b**) iSLK cells (iSLK-WT, iSLK-21KD, and iSLK-21del) were cultured for 48 h in a medium with Dox and NaB, and the culture supernatants were harvested. KSHV genomes were purified from the cell-free genome-encapsidated particles in culture supernatants, and viral DNA copies were determined by real-time PCR. N.S., not significant.

To examine the contribution of ORF21 on the extracellular production of particles containing viral genomes in KSHV lytic replication, the DNA copies of viral genome-encapsidated particles in the culture supernatant were evaluated. iVero-WT, iVero-21KD, and iVero-21del cell lines were treated with Dox and NaB for 48 h, and the culture supernatants were harvested. The encapsidated KSHV genome in the viral particles was prepared from culture supernatants and quantified by qPCR. The data showed that the production of viral particles was comparable among iVero-WT, iVero-21KD, and iVero-21del cells (Figure 4a). In addition to iVero cells, the production of viral particles in iSLK-WT, -21KD, and -21del cells was also nearly equal. The results for iVero (and iSLK)-21KD cells indicate that ORF21-kinase function did not affect virus production, which was consistent with the report by Beauclair et al. [18]. This means that neither the ORF21-kinase activity nor the ORF21 molecule itself is associated with the extracellular production of genome-encapsidated particles in lytic replication.

### 2.5. ORF21 Is Involved in the Infectivity Enhancement of the Progeny Virus

**Figure 5:**
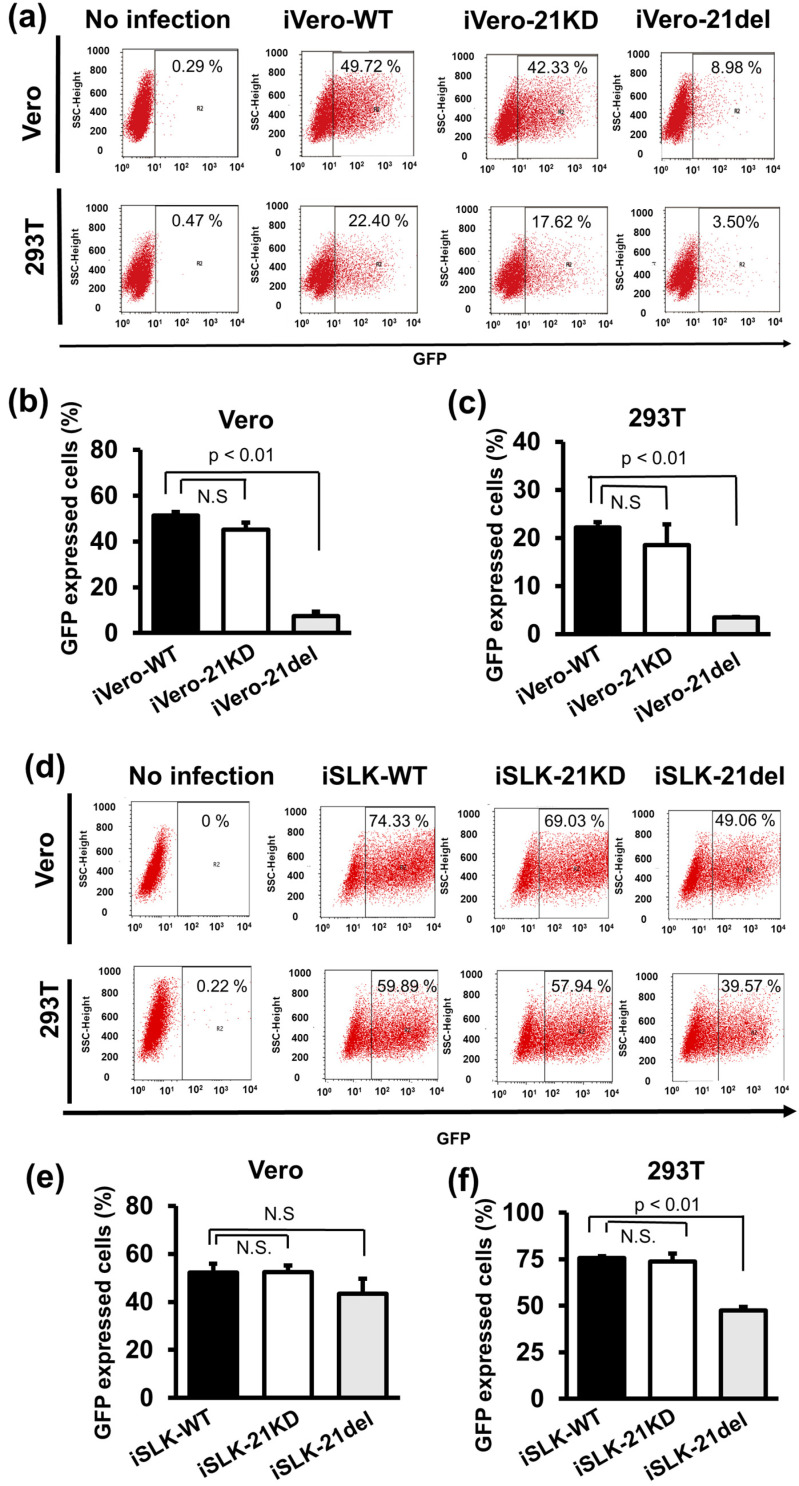
ORF21 is involved in infectious virus production. (**a**–**c**) Recombinant BAC16-harboring iVero cells (iVero-WT, iVero-21KD, and iVero-21del) or (**d**–**f**) iSLK cells (iSLK-WT, iSLK-21KD, and iSLK-21del) were treated for 96 h with Dox and NaB to produce recombinant KSHV, and the culture supernatants were harvested. The progeny viral particles were precipitated with ultracentrifugation, and partially purified viral particles (at approximately 10^5^ viral genome copies/cell) were used to infect fresh Vero cells (**b**,**e**) and 293T cells (**c**,**f**). The GFP-positive cells (i.e., infected cells) were counted by flow cytometry 48 h post-infection to determine the infectivity of the produced recombinant viruses. N.S., not significant. *p* < 0.01 indicates a statistically significant difference compared with the iVero/iSLK-WT cells.

Because ORF21 was not essential for the extracellular production of viral genome-encapsidated particles, we focused on the production of infectious virions (that is, the infectivity of the produced virus) as the ORF21 function. Therefore, the effects of the ORF21-defects on the production of infectious virions were elucidated. To determine the infectivity of recombinant KSHV through the infectivity assay, WT, ORF21-kinase dead, and ORF21-deleted KSHV were produced from iVero-WT, iVero-21KD, and iVero-21del cells, respectively. The BAC16-harboring cells were treated for 96 h with Dox and NaB, and viral particles in the culture supernatant were precipitated by ultracentrifugation. Prepared viral particles were infected into Vero and 293T cells, and the cells were cultured for 48 h. The infected cells (BAC16-derived GFP-positive cells) were analyzed using flow cytometry (Figure 5a–c). The same experiments were performed using iSLK cells (Figure 5d–f). In the case of recombinant KSHV produced from iVero cells, a significant decrease in the infectivity of ORF21-deleted KSHV in both Vero and 293T cells was observed compared with ORF21-kinase dead KSHV and WT KSHV. In the case of recombinant KSHV from iSLK cells, a significant decrease in the infectivity of ORF21-deleted KSHV was observed only in the 293T cells (Figure 5e,f), although the infectivity of the ORF21-deleted KSHV in both the Vero and 293T cells was lower than the infectivity of ORF21-kinase dead KSHV and WT KSHV. This may have been due to a difference in the sensitivities of Vero and 293T cells to virus infection. However, the infectivity of the produced recombinant viruses was unchanged between ORF21-kinase dead KSHV and WT KSHV, which is similar to the results reported by Beauclair et al. [18].

**Figure 6:**
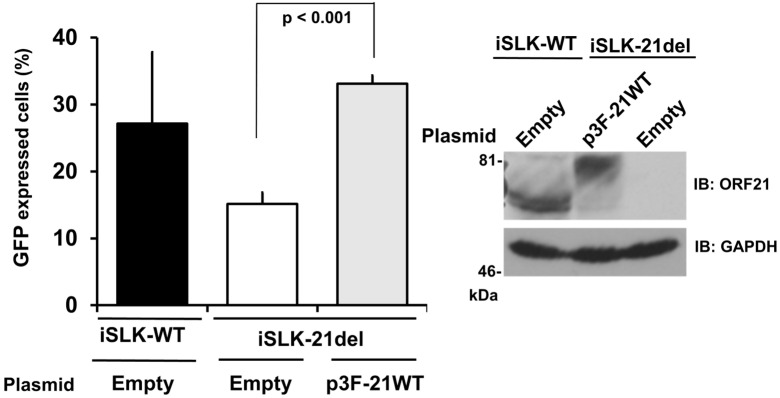
Infectious virus production in iSLK-21del cells is rescued by ORF21 overexpression. To validate the rescue of infectious virus production in iSLK-21del cells by exogenous ORF21 expression, iSLK-21del cells were transiently transfected with the 3×Flag-ORF21 wildtype (p3F-21WT) plasmid (or empty plasmid) and treated for 96 h with Dox and NaB to produce recombinant KSHV. The culture supernatant was ultracentrifuged to precipitate the produced viruses, which were inoculated onto 293T cells. The infected cells (GFP-positive cells) were analyzed using a flow-cytometer, and the infectivity of the recombinant viruses is shown as a bar graph. *p* < 0.001 indicates a statistically significant difference compared with the empty plasmid-transfected cells. The endogenous or exogenenous ORF21 expression was analyzed by Western blotting using the anti-ORF21 polyclonal antibody. The original images are shown in Supplementary Figure S3.

To gain further evidence regarding the effect of ORF21 on infectious virus production, a complement assay using exogenous ORF21 expression in ORF21-deficient KSHV-harboring cells was performed. The flag-tagged ORF21 plasmid was transiently transfected into the iSLK-21del cell line, and virus production was induced by Dox- and NaB-treatment. The infectivity of the produced virions was evaluated by an infectivity assay. As a result, the infectivity of virus from iSLK-21del cells was lower than that from iSLK-WT. However, the infectivity of the virus from the iSLK-21del cells was recovered significantly when the Flag-ORF21 plasmid was exogenously overexpressed (Figure 6). Figure 5 and Figure 6 show that the infectivity of the ORF21-deleted KSHV was lower than that of the WT KSHV and ORF21-kinase dead KSHV. However, the infectivity of ORF21-kinase dead KSHV was almost equal to that of WT KSHV. This indicates that the infectivity retention of the produced virions did not require the kinase activity of ORF21, but the expression of the ORF21 molecule itself. Moreover, an unknown ORF21 molecule-dependent function (other than the kinase activity of ORF21) was thought to be involved in the infectivity of the progeny virus (or the achievement of effective infection).

### 2.6. ORF21 Upregulates MEK Phosphorylation and Anchorage-Independent Cell Growth

**Figure 7:**
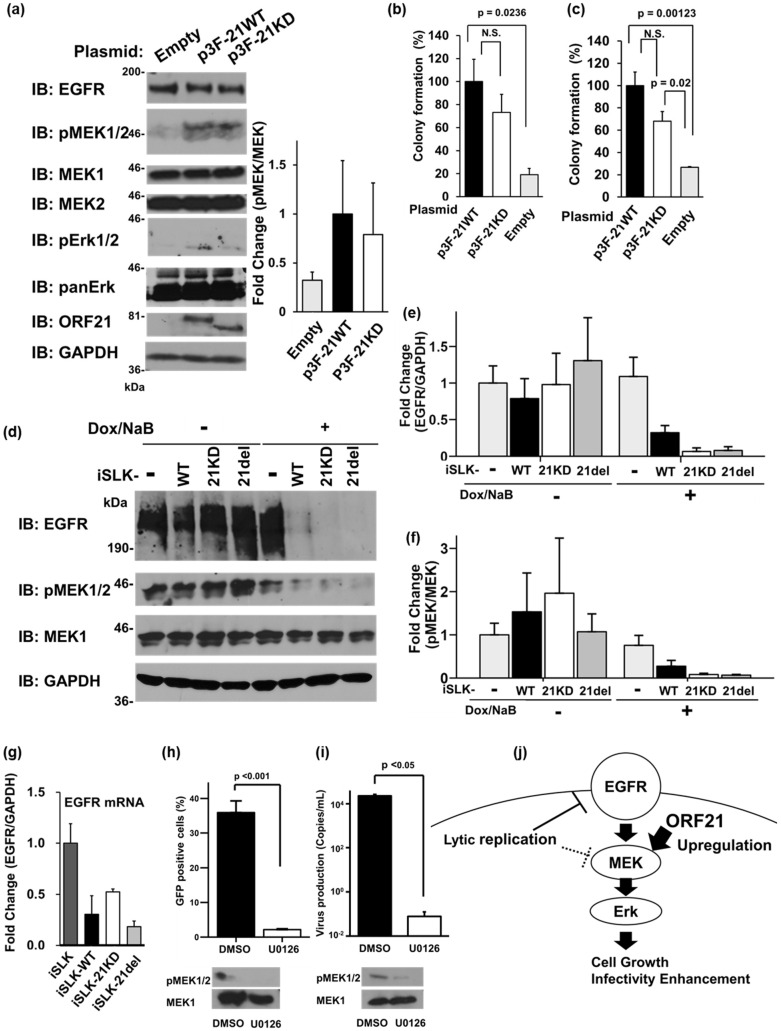
ORF21 upregulated the MEK phosphorylation and anchorage-independent cell growth. (**a**–**c**) Upregulation of MEK phosphorylation, anchorage-dependent proliferation, and anchorage-independent proliferation by ORF21. HeLa cells were transfected with empty, 3×Flag-ORF21 wildtype (p3F-21WT), or 3×Flag-ORF21-kinase dead (p3F-21KD) plasmid and cultured for 48 h, followed by (**a**) Western blotting, (**b**) cell proliferation assay, or (**c**) soft agar colony formation assay. (**a**) The band intensities of phospho-MEK were calculated using Fiji software. The values of phospho-MEK/total MEK are presented as a bar graph. The original images of phospho-MEK and Erk are shown in Supplementary Figure S4a. (**b**,**c**) The value of 21WT plasmid-transfected cells is presented as 100. *P*-values indicate a significant difference. N.S., not significant. (**d**–**f**) KSHV lytic replication downregulated the MEK phosphorylation and the expression of the EGF receptor (EGFR). iSLK (without BAC16), iSLK-WT, iSLK-21KD, and iSLK-21del cells were treated for 48 h with Dox and NaB to express the lytic-related viral proteins, and EGFR and the phosphorylated MEK were analyzed. iSLK cells (without BAC16) were used as the uninfected control. The original images are shown in Supplementary Figure S4b. (**e**,**f**) The values of EGFR/GAPDH, and phosphorylated MEK/MEK are presented as a bar graph. The values of Dox- and NaB-untreated iSLK cells were defined as 1.0. (**g**) Expression of EGFR mRNA in the lytic-induced iSLK-WT, iSLK-21KD, and iSLK-21del cells. Cells were treated with Dox and NaB for 48 h, and the expression of EGFR mRNA was determined by RT-qPCR and normalized by the expression of GAPDH mRNA. (**h**) The effect of the MEK-signaling inhibitor (U0126) on the new infection of the progeny virus with the recipient cells. The recipient (293T) cells were pretreated with 5 μM U0126 or control DMSO for 48 h, and WT-KSHV particles were infected into U0126-pretreated 293T cells. The number of infected cells (GFP-positive cells) was analyzed by flow cytometry. The inhibition of MEK signaling was validated by Western blotting. (**i**) The effect of U0126 on viral production in the lytic-induced WT-BAC16-harboring cells. The WT-BAC16-harboring cells were treated with Dox and NaB in the presence of 100 μM U0126 for 48 h, and the culture supernatant was harvested. The number of produced viruses in the culture supernatant was measured by qPCR. The inhibition of MEK signaling was validated by Western blotting. (**h**,**i**) N.S., not significant. *p* < 0.05 and *p* < 0.001 indicate a statistically significant difference. (**j**) Model of the KSHV ORF21-mediated upregulation of the MEK pathway and cell growth. The EGFR-MEK signaling pathway is known to be necessary for anchorage-independent cell growth in tumor cells. Unknown KSHV lytic-related proteins expressed by the lytic reactivation suppresse the expression of EGFR and MEK phosphorylation. ORF21 upregulates the MEK phosphorylation, resulting in enhancement of the infectivity of the progeny virus and anchorage-independent cell growth.

An ORF21 molecule-dependent function was involved in the enhancement of the infectivity of the produced virus (Figure 5 and Figure 6). ORF21 induced the shrinkage of host cells under KSHV lytic infection (Figure 2). Moreover, ORF21 has been reported to affect the signaling pathways involved in immune response suppression, cell contraction, and cell migration [17,19]. To gain further insight into the molecule-dependent and kinase activity-dependent ORF21 functions of ORF21 within host cells, we analyzed the effects of exogenous and endogenous ORF21 expression on several signaling pathways. As a result, although NF-κB, AKT, STAT, p38MAPK, JNK, Wnt/b-catenin, p53, and so on were not influenced by the ORF21 expression, MEK phosphorylation was upregulated by the ORF21 expression (Figure 7). HeLa cells were transiently transfected with Flag-ORF21 (p3F-21WT) or Flag-ORF21-kinase dead (p3F-21KD) plasmid, and, at 48 h post-transfection, the cells were analyzed by Western blotting. The phosphorylation level of MEK was markedly increased in p3F-21WT- and p3F-21KD-transfected cells compared with the empty plasmid-transfected cells (Figure 7a). Furthermore, the phosphorylation of ERK, a downstream substrate of MEK, was somewhat enhanced in p3F-21WT and p3F-21KD-transfected cells. Because MEK signaling is known to enhance cell proliferation, we studied the influence of ORF21 on anchorage-dependent and -independent cell growth. HeLa cells transfected with empty, p3F-21WT, or p3F-21KD plasmid were cultured on a 96-well plastic plate for 48 h, and the cell numbers were analyzed for anchorage-dependent cell growth using a proliferation assay (Figure 7b). Similarly, transfected HeLa cells were cultured in soft agar containing DMEM for 8 days, and the formed colonies were evaluated for anchorage-independent cell growth (Figure 7c). As expected, exogenous ORF21 and ORF21-KD expression enhanced both the anchorage-dependent and -independent growth. Moreover, we analyzed the effects of ORF21 and ORF21-KD endogenously expressed by lytic replication on MEK phosphorylation in mutated BAC16-harboring cells. iSLK-WT, iSLK-21KD, and iSLK-21del cells were treated with Dox and NaB to induce lytic replication, and we analyzed not only the phosphorylated MEK, but also the EGF receptor (EGFR), which is the uppermost signal-receptor of MEK signaling. Interestingly, phosphorylated MEK and EGFR were downregulated in all lytic-induced KSHV-harboring cells (Figure 7d). As for iSLK-WT and iSLK-21KD cells, some phosphorylated MEK remained compared with the iSLK-21del cells. It is thought that some phosphorylated MEK was observed in lytic-induced iSLK cells because lytic-induced ORF21 and ORF21-KD expression caused MEK phosphorylation. Over 60 kinds of lytic-related viral molecules were expressed in KSHV-infected cells at the lytic phase. Therefore, an unknown lytic-related molecule(s), other than ORF21, downregulated the EGFR expression as well as the phosphorylation of MEK in lytic-induced iSLK-WT, iSLK-21KD, and iSLK-21del cells. Next, in order to know whether the lytic-induced downregulation of EGFR was due to its transcriptional inactivation, we examined the expression level of EGFR mRNA. Transcription of those molecules in lytic-induced iSLK-WT, iSLK-21KD, and iSLK-21del cells was reduced compared with that in uninfected iSLK cells. This indicated that the EGFR expression was transcriptionally downregulated in KSHV-infected cells in the lytic phase (Figure 7g). 

ORF21-WT and ORF21-kinase dead enhanced the infectivity of the progeny virus (Figure 5) and also upregulated MEK signaling (Figure 7a). Therefore, we can infer that ORF21 localized as a tegment protein in a virion is transmitted into the recipient target cell (i.e., host cell), and transmitted ORF21 enhances the infection through MEK upregulation within the recipient cell. To examine whether MEK upregulation contributed the establishment of the novel infection of the progeny virus, we analyzed the effects of the MEK-signaling inhibitor (U0126) on the infection of the progeny virus with 293T cells. The recombinant WT-KSHV particles were prepared by ultracentrifugation from the culture supernatant of Dox and NaB-treated WT-BAC16-harboring cells. The recipient (293T) cells were pretreated with 5 μM U0126 or control DMSO for 48 h, and WT-KSHV particles were infected into U0126-pretreated 293T cells. The cells were cultured for 48 h, and the number of infected cells (GFP-positive cells) was analyzed by flow cytometry (Figure 7h). The results showed that MEK inhibition by U0126 suppressed the number of progeny virus-infected cells. These suggest that ORF21 transmitted as a tegument protein enhances the infectivity of the virus through MEK upregulation in the recipient cell. On the other hand, we can also infer that ORF21 expressed as a lytic viral protein enhances the production of the infectious progeny virus through MEK upregulation within the infected cell at the lytic phase. To examine whether MEK upregulation contributed to viral production, we analyzed the effects of U0126 on the production of viral particles in the lytic-induced infected cells. The WT-BAC16-harboring iSLK cells were treated with Dox and NaB in the presence of 100 μM U0126, and the produced viruses were evaluated by the qPCR quantification of the encapsidated KSHV genome in the culture supernatant. As WT-BAC16-harboring iSLK cells were resistant to U0126, 100 μM U0126 was necessary for inhibition of the MEK signaling. The data showed that U0126 treatment reduced virus production, which is in good agreement with the previous reports [18,21]. This suggests that ORF21 upregulates MEK signaling, resulting in an enhancement of not only the infectivity of the progeny virus with the recipient cell, but also the production of progeny viruses in the lytic-induced cell. We confirmed that the treatment with 100 μM U0126 did not affect the cell viability of iSLK. However, we need to consider that several cellular functions including kinases may be affected by high concentration of U0126.

## 3. Discussion

We analyzed ORF21-kinase dead KSHV (21KD), ORF21-deleted KSHV (21del), and their harboring cells using the anti-ORF21 rabbit polyclonal antibody. ORF21 was expressed at 30~36 h after lytic induction (Figure 2a). It was localized in the whole cytoplasm and induced cell contraction during the lytic infection (Figure 2b–d). The ORF21-kinase function as well as the ORF21 molecule itself were not important for the transcription of lytic genes, viral genome replication, or the production of genome-encapsidated particles; however, an unknown ORF21 molecule-dependent function(s), other than the kinase function of ORF21, was involved in the efficiency of the progeny virus infection (Figure 5 and Figure 6). Transient ORF21 expression increased the amount of phosphorylated MEK and the colony-forming ability (i.e., anchorage-independent cell growth) (Figure 7a,c). Although phosphorylated MEK and EGFR were markedly downregulated in lytic-induced KSHV-harboring cells, some EGFR and phosphorylated MEK remained in WT KSHV-harboring cells compared with ORF21-mutated (21KD and 21del) harboring cells (Figure 7d). MEK inhibition by U0126 suppressed the novel infection of the progeny virus with the recipient 293T cells (Figure 7h). Figure 7j shows the manipulation of the EGFR–MEK signaling pathway by ORF21 during KSHV lytic replication. KSHV lytic replication suppressed the EGFR–MEK signaling pathway through the downregulation of EGFR and phosphorylated MEK. An unknown ORF21 molecule-dependent function(s) promoted the phosphorylation of MEK, resulting in the upregulation of EGFR–MEK signaling and, subsequently, anchorage-independent cell growth. 

Previous studies have shown that unlike the TK activity of HSV-1 UL23, the TK activity of KSHV ORF21 is not potent and does not contribute to viral replication and virus production [11,12,18]. These features of ORF21 are consistent with the findings in the ortholog (BXLF1) of EBV, which belongs to the same gammaherpesvirus subfamily as KSHV [22]. As for the ORF21/TK ortholog of murine gammaherpesvirus 68 (MHV-68), which belongs to the same Rhadinovirus genus of the gammaherpesvirus subfamily as KSHV, the ORF21/TK gene-disrupted MHV-68 mutant can replicate normally in vitro; however, the amount of infectious virus or the infectivity of virus is decreased in ORF21/TK gene-disrupted MHV-68 compared to that of WT MHV-68 [23,24,25]. Thus, it is conceivable that the ORF21/TK orthologs of the gammaherpesvirus subfamily do not affect virus production or viral genome replication in vitro, but they may affect the infectivity of the progeny virus. Furthermore, when mice were infected with ORF21/TK-knockout MHV-68, this knockout MHV-68 had a lower infectivity than WT MHV-68, but could still migrate to the target lymphoid tissues [25]. Taken together with our results and those for ORF21/TK orthologs of the gammaherpesvirus, KSHV ORF21 is thought to contribute to the new infection through three mechanisms: (i) enhancement of infectivity of progeny virus, (ii) establishment of novel infection in the host cell after invasion into the cell, and (iii) the spread of the virus by promoting the migration of infected cells. 

Regarding the ORF21-mediated infectivity enhancement of the progeny virus, the interaction of ORF21 as a tegument protein with ORF64 (tegument protein) and gN (envelope protein) is thought to be responsible for the enhanced infectivity. The MS analysis of KSHV virions showed that ORF21 was a component protein of the virion [9,26,27]. In addition, Zhu et al. reported that ORF21 was a tegument protein binding to the nucleocapsid [9]. Rosen et al. found that ORF21 formed a complex with ORF64 and gN in the co-immunoprecipitation assays [28]. Given these reports and our data showing a decrease in the infectivity of the virion by ORF21 molecule-deletion (Figure 5 and Figure 6), we speculate that ORF21 is involved in infectivity enhancement via an interaction with ORF64 and other ORF protein(s) within the progeny virus. In the future, research into the transition process from a nucleocapsid to a matured virion, and the contribution of ORF21 to this process will help to elucidate one aspect of the formation of this highly infectious virus. 

Regarding the involvement of ORF21 in establishing a novel infection, we speculate that a nucleocapsid, along with tegument proteins including ORF21, is released from the virion into the cell after virus-binding to the host cell. Then, the released ORF21 protein may upregulate the MEK signaling pathway, resulting in the achievement of effective infection. In fact, the MEK signaling pathway is known to be upregulated by novel KSHV infection [29,30]. Moreover, MEK upregulation was reported to be necessary for not only the new infection of a wildtype virus derived from BCBL1 PEL with a human foreskin fibroblasts cell [31], but also the production of a progeny virus in rKSHV.219-harboring cells [21]. Furthermore, our data showed an increase in phosphorylated MEK (i.e., upregulation of the MEK pathway) through endogenous and exogenous ORF21 expression. The pretreatment with the MEK inhibitor U0126 in the recipient cells suppressed the new infection of the progeny virus with the target cells (Figure 7h). Thus, it is conceivable that ORF21, one of the tegument proteins, is released from the virion and may contribute to the establishment of novel infection through MEK upregulation in the host cell.

Regarding the ORF21-mediated virus spread, ORF21 was involved in the activation of RhoA–ROCK signaling and cell contraction, and Gill et al. inferred that ORF21 contributes to the spread of KSHV infection by promoting the migration of infected cells [17]. Namely, it is thought that ORF21 promotes the spread of KSHV-infected cells, resulting in long-distance transmission. The migrated virus along with infected cells can establish a novel infection at distant sites by cell-to-cell infection or cell-free infection. Our data showed the cytoplasmic localization of endogenous ORF21 and a cell contraction by ORF21 (Figure 2b–d), which are consistent with previous reports [14,17]. We also showed that ORF21 mediated the upregulation of the MEK pathway and anchorage-independent cell growth (Figure 7). Although RhoA–ROCK signaling is well known to promote cell migration [32,33], the MEK pathway has also been reported to contribute to the promotion of cell migration [34,35,36,37]. 

A previous study reported that ORF21 suppressed TLR2, TLR4, and NF-κB signaling [19]. Moreover, in a comprehensive analysis of the interactions between KSHV-encoded proteins and host cellular proteins, ORF21 was reported to interact with the proteins involved in RNA processing, phosphorylation regulation, and RNA metabolism [38]. In other viral orthologs of KSHV ORF21, an interaction with the centrosome has been reported in EBV BXLF1 [39]. BXLF1 was localized at the centrosome and surrounded the tubulin-rich central core in a microtubule-independent manner. Viral thymidine kinase-deleted MHV-68 normally replicates viral DNA in the spleen and peritoneal cells; however, the infectivity of mutated MHV-68 was markedly decreased in those cells compared with WT [25]. Thus, it is interesting that viral thymidine kinase-deleted MHV-68 failed to establish a new infection, which is in good agreement with the properties of KSHV ORF21. This may be because KSHV and MHV-68 belong to the same genus in the gammaherpesviruses subfamily. Although there are differences in the functions of viral thymidine kinases among this viral subfamily, it is conceivable that ORF21 orthologs of human herpesviruses would affect the cellular signaling pathway, cytoskeletal system, and cytoplasmic proteins, resulting in the establishment of a controlled viral life cycle and effective infection.

In recent years, new conceptions such as biological phase separation and liquid–liquid phase separation (LLPS) have been the focus of the life science field [40,41,42]. Until now, intracellular proteins have been assumed to be in a homogeneous dilute solution, and based on that assumption, intracellular protein–protein interactions and enzymatic reactions have been studied. Regarding the LLPS conception, cellular proteins (also DNA) exist in either a crowded or a diluted condition. The phase separation in LLPS occurs by the concentration difference between the high- and low-concentration of the biomolecule. LLPS can regulate various biological reactions, such as phosphorylation, through changes in the phase separation [43,44,45]. Our results indicate the importance of the ORF21 molecule itself rather than its function as a kinase. As for the other functions of ORF21 (other than the kinase), we can speculate a scaffold function is involved in the protein–protein interactions. Perhaps under the special condition of cell infection, ORF21 distributed in the cytoplasm can cause crowding spots of ORF21 and the LLPS formation of ORF21, which in turn exert an ORF21 molecule-dependent function (e.g., MEK phosphorylation). Because the concentration and distribution of cellular and viral proteins are significantly changed by the viral infection and viral life cycle, biological analysis based on the LLPS concept may be needed in the future as a more effective research method.

## 4. Material and Methods

### 4.1. Plasmids

To construct the 3×Flag-ORF21-wildtype plasmid (p3F-21WT), the KSHV ORF21 coding DNA fragment was obtained by PCR from HBL6 PEL cells [46] and was subcloned into a 3×Flag-tagged pCI-neo vector, which was generated by inserting oligonucleotides encoding three repeats of the Flag-tag sequence into the pCI-neo mammalian expression vector (Promega). To construct the 3×Flag-ORF21-kinase dead plasmid (p3F-21KD), the KD mutation (Gly260Val, Gly263Val, and Gly265Val) of the ORF21 gene was generated by overlap extension PCR with KOD-Plus Neo (Toyobo, Osaka, Japan) using the 3xFlag-ORF21 plasmid as the template. The inserted sequences were verified by Sanger sequencing. All of the primers used for the construction of the expression plasmids are shown in Table 1.

### 4.2. Preparation of Anti-ORF21 Rabbit Polyclonal Antibody

To construct glutathione S-transferase (GST)-tagged ORF21, KSHV genome-including cellular genomic DNA was purified from HBL6 cells [46] using the QIAamp DNA blood mini kit (Qiagen, CA, USA). The ORF21-coding DNA sequence was amplified by PCR from the purified DNA. Amplified ORF21 DNA was cloned into the pGEX-6P-1 bacterial expression vector (GE Healthcare, Chicago, IL, USA), which had a cleavage site of PreScission protease between GST and the multi-cloning site. *E. coli* cells (BL21 strain) were transformed with the GST-tagged ORF21 plasmid, and the GST-tagged ORF21 protein was adsorbed onto Glutathione-Sepharose 4B beads. The ORF21 protein was cleaved from GST adsorbed on the glutathione beads by PreScission protease, and the eluted ORF21 protein was collected and used as an immunogen. Immunizations of rabbits with purified ORF21 protein, blood collection, and blood serum preparation were commercially performed by Protein Purify Co. (Gunma, Japan). Anti-ORF21 polyclonal antibodies were purified with the blood serum with affinity-chromatography using GST- ORF21 protein-binding Glutathione-Sepharose 4B and stored at −20 °C.

### 4.3. Mutagenesis of KSHV BAC16

KSHV bacterial artificial chromosome clone 16 (BAC16) established by Brulois et al. [20] was used to construct ORF21-kinase dead KSHV BAC16 (21KD) and ORF21-deleted KSHV BAC16 (21del), and the mutagenesis of KSHV BAC16 was performed according to their method [20]. The primer sequences used for mutagenesis are noted in Table 1. The insertion and deletion of kanamycin resistance cassettes (Kan^R^) in each mutant were analyzed by digestion with Hind III and agarose gel electrophoresis. The mutated sites of each BAC clone were confirmed by Sanger sequencing.

### 4.4. Establishment of Doxycycline-Inducible Recombinant KSHV-Expressing Cells

To establish efficient recombinant virus-producing cells, tetracycline/doxycycline (Dox)-inducible (Tet-On) RTA/ORF50-expressing iSLK cells [47,48,49] or iVero cells [50] were used as recombinant KSHV-producing cells. For maintenance, iSLK cells were cultured in a growth medium containing 1 μg/mL of puromycin (InvivoGen, CA, USA) and 0.25 mg/mL of G418 (Nacalai Tesque, Kyoto, Japan). iVero cells were cultured in a growth medium containing 2.5 μg/mL of puromycin. WT KSHV BAC16 (WT-BAC16) and ORF21-mutated KSHV BAC16 (21KD-BAC16 and 21del-BAC16) were transfected into iSLK and iVero cells by the calcium phosphate method [51]. The transfected cells were selected under 1000 μg/mL of hygromycin B (Wako, Osaka, Japan) to establish doxycycline-inducible recombinant KSHV-producing cell lines. iSLK (or iVero) cells transfected with WT-BAC16, 21KD-BAC16, and 21del-BAC16 were named iSLK-WT, iSLK-21KD, and iSLK-21del cells, respectively (or iVero-WT, iVero-21KD, and iVero-21del cells, respectively). To induce the lytic replication for recombinant KSHV production, BAC16-harboring iSLK (or iVero) cells were treated with sodium butyrate (NaB) and Dox. NaB binds to histone deacetylases (HDACs) and induces the hyperacetylation of the histones, resulting in transcriptional activation. Dual treatment of both Dox and NaB was used in this study because it induced the RTA/ORF50 expression and the lytic cycle more effectively.

### 4.5. Measurement of Extracellular Viral Genome-Encapsidated Particles and Intracellular Viral DNA Replication

BAC16-transfected iSLK cells and BAC16-transfected iVero cells were treated with 1.5 mM NaB and 8 μg/mL Dox for 48 h to induce lytic replication and the production of recombinant KSHV. For the quantification of the extracellular viral genome-encapsidated particle production in the culture supernatant, the culture supernatants of the lytic-induced cells were harvested, and the culture supernatants (220 μL) were treated with four units of DNase I (New England Biolabs, MA, USA) to obtain only enveloped and encapsidated viral genomes. Viral DNA was purified and extracted from 200 μL of the DNase I-treated culture supernatant using a QIAamp DNA blood mini kit (Qiagen). To quantify the intracellular viral DNA copy number, SYBR green real-time PCR was performed using KSHV-encoded ORF11-specific primers, as shown in Table 1. For measurement of the KSHV genome replication, BAC16-transfected iSLK cells were treated with Dox and NaB for 48 h, and the total cellular DNA containing the KSHV genome DNA was purified from harvested cells using the QIAamp DNA blood mini kit (Qiagen). The number of cellular KSHV genome copies was determined by SYBR green real-time PCR and normalized to the total number of DNA copies.

### 4.6. RT Real-Time PCR (RT-qPCR)

mRNA was extracted from the cells using RNAiso Plus (Takara Bio, Osaka, Japan). cDNA was synthesized using a ReverTra Ace qPCR kit (Toyobo) and subjected to SYBR green real-time PCR. The sequences of the RT-qPCR primer sets had been previously described [50], as shown in Table 1. Relative mRNA expression levels were determined by the GAPDH expression and ΔΔCt methods.

### 4.7. Infectivity Assay

The infectivity titer of the produced recombinant virus in the culture supernatant was evaluated by an infectivity assay [20]. iSLK (or iVero) cells harboring the mutated BAC16 were treated with 8 μg/mL Dox and 1.5 mM NaB for 96 h, and the culture supernatants were harvested. The supernatants were passed through a 0.45 um filter and ultra-centrifuged. Precipitates containing the virus were suspended in culture media. The genome number of concentrated viruses was quantified using qPCR. Concentrated viruses (at approximately 10^5^ viral genome copies/cell) were inoculated onto Vero or HEK293T cells in the presence of 8 μg/mL polybrene (Sigma-Aldrich, MO, USA). After 48 h, infectivity (GFP-positive cells) was analyzed using a FACS Calibur (Becton Dickinson, CA, USA).

### 4.8. Complementation Assay

The complementation assay was performed as previously described, but with several modifications [49,52]. The iSLK-21del cells (1 × 10^6^ cells) were transfected with the 3×Flag-ORF21 plasmid using ScreenFect A plus (Wako) and treated with Dox and NaB. After 96 h, the culture supernatant including the viruses was collected and centrifuged at 15,000 rpm for 10 min at room temperature, and then the supernatant (1000 μL) was mixed with 1 × 10^6^ trypsinized HEK293T cells. The cell–virus mixture was incubated with 8 μg/mL polybrene (Sigma-Aldrich) and was placed into a 12-well plate or 24-well plate. The 12-well plate or 24-well plate containing cells was centrifuged at 1200 × g for 1 h at room temperature for effective infection, and the cells were cultured at 37 °C for 24 h. GFP-positive cells (i.e., infected cells) were analyzed using a FACS Calibur (Becton Dickinson).

### 4.9. Agents, Cell Culture and Western Blot

U0126 was obtained from FUJIFILM Wako Pure Chemical Co. (Osaka, Japan) and dissolved in dimethyl sulfoxide (DMSO). HEK293T, Vero, and HeLa cells were cultured in DMEM supplemented with 5% fetal calf serum (FCS). iSLK cells were cultured in DMEM containing 5% FCS, 1 μg/mL puromycin (InvivoGen), and 0.25 mg/mL G418 (Nacalai Tesque). iVero cells were cultured in DMEM containing 5% FCS and 2.5 μg/mL puromycin. For the Western blot analysis [47], the cells were lysed with an SDS sample buffer (containing 1% 2-mercaptoethanol, 0.1 mM NaF, 1 mM β-glycerophosphate, 1 µg/mL aprotinin, 1 µg/mL pepstatin, and 0.25 mM PMSF) and sonicated [53,54]. Anti-EGFR, anti-phosphorylated MEK (Ser217/221) (Cell Signaling Technology, MA, USA), anti-MEK1, anti-MEK2, anti-phosphorylated-Erk1/2, pan-Erk (Becton Dickinson), and anti-GAPDH (Santa-Cruz, CA, USA) were used as the primary antibody. Horseradish peroxidase (HRP)-linked anti-mouse or anti-rabbit IgG antibody (GE Healthcare) was used as the secondary antibody. The band intensities of the proteins detected in the Western blot were measured using Fiji software (ver1.53.q, NIH, Bethesda, MD, USA) [55].

### 4.10. Immunofluorescence assay (IFA) and Evaluation of Cell Contraction

Immunofluorescence assays were performed as described previously [47,48,50,52]. The cells seeded onto a six-well plate on a glass slide were fixed with 4% paraformaldehyde at room temperature for 10 min (Figure 2c,d) or with 4% paraformaldehyde at room temperature for 10 min and 50% acetone in methanol (Figure 2b). The fixed cells were permeabilized with 0.1% Triton X-100 in PBS. The samples were treated with 1.5% FCS in PBS and incubated overnight with primary antibodies at 4 °C. After washing with PBS-T (0.5% FCS and 0.05% Tween 20 in PBS), the cells were incubated with the secondary antibody Alexa Fluor 568 conjugated anti-rabbit IgG (InvivoGen) for 1 h at 37 ºC and were washed with PBS-T. The stained samples were embedded in Fluoro-KEEPER Antifade Reagent non-hardening type with DAPI (Nacalai Tesque) and observed under a fluorescence microscope (Olympus IX71) (Olympus, Tokyo, Japan) and a confocal LSM 800 microscope (Carl Zeiss, Oberkochen, Germany) using DP2-BSW (Olympus) and LSM software (Carl Zeiss), respectively.

As for the evaluation of the cell contraction, iSLK cells were stained with Phalloidin CruzFluor™ 633 Conjugate (Santa-Cruz), and the GFP-positive cells (i.e., BAC16-harboring cells) were analyzed using ImageJ software (ver1.52a, NIH, Bethesda, MD, USA) and Fiji (ver1.53.q, NIH, Bethesda, MD, USA) [55,56]. The cell border lines indicated by the phalloidin and GFP signal were edged on the software, and each area of the edged cells was measured. The cumulative percentage of cells in the quantified area was calculated, and statistical analysis was performed after removing the top and bottom 5 (%) of the total cumulative percentage (=total 90%).

### 4.11. Two-Layered Soft Agar Colony Formation Assay

The two-layered soft agar colony formation assay was carried out as previously described mixed, but with a few modifications [53]. The transfected HeLa cells were mixed with the upper layer medium (DMEM containing 0.35% agarose and 20% FCS), heated at 50 °C, and seeded in the well of a 12-well plate coated with the bottom layer medium (DMEM containing 0.5% agar and 20% FCS). The cells were cultured in agar-containing the upper layer medium for 8 days. The colonies were visualized using a fluorescence microscope (Olympus IX71), and the number of GFP-positive colonies was counted.

### 4.12. Cell Proliferation Assay

Transfected HeLa cells were seeded in 96-well plates and cultured in DMEM supplemented with 5% FCS for 2 days. The viable cell number was measured using Cell Count Reagent SF (Nacalai Tesque). The optical density at 450 nm for each sample was measured with a spectrophotometer (Tecan M200; Tecan, Kanagawa, Japan).

### 4.13. Statistical Analysis

Microsoft Excel^®^ and R (version 4.2.1, R Foundation for Statistical Computing, Vienna, Austria) with R Studio were used for the statistical analysis. Welch’s *t*-test was used for the comparison of two groups. In the case of multiple comparisons, *t*-tests with the Holm–Bonferroni method, Dunnett tests, or Tukey–Kramer’s tests were used.

## Figures and Tables

**Table 1:** Primers for BAC mutagenesis, construction of expression plasmids, real-time PCR, and RT real-time PCR.

Primer_Discription Direction	Sequence (5’ -> 3’)
**[BAC mutagenesis] *^a^***	
**KSHV ORF21-Kinase Dead Forward**	accgtggactacaggaatgtttatttgctttacttagag- GTTgtaatg-GTAgtgGTCaaatcaacgctggtcaacgTAGGGATAACAGGGTAATCGATTT
**KSHV ORF21-Kinase Dead Reverse**	gggcaagatcccgcacacggcgttgaccagcgttgattt- GACcacTACcattacAACctctaagtaaagcaaataaGCCAGTGTTACAACCAATTAACC
**KSHV ORF21-Knock Out Forward**	gtcagcgactgacgacgactcgggagactacgcgccaatg- TAGTTAGATAGTgatcgcttcgccttccagagTAGGGATAACAGGGTAATCGATTT
**KSHV ORF21-Knock Out Reverse**	ggcgaccacacaccctggggctctggaaggcgaagcgatc- ACTATCTAACTAcattggcgcgtagtctcccgGCCAGTGTTACAACCAATTAACC
**[plasmid] *^b^***	
**ORF21 KD Forward**	gtggactacaggaatgtttatttgctttacttagagGTTgtaatgGTAgtgGTCaaatcaacgctggtcaacgccg
**ORF21 KD Reverse**	cggcgttgaccagcgttgatttGACcacTACcattacAACctctaagtaaagcaaataaacattcctgtagtccac
**[Real-time PCR]**	
**ORF11-qPCR Forward**	TTGACAACACGCACCGCAAG
**ORF11-qPCR Reverse**	AAAAATCAGCACGCTCGAGGAG
**[RT-real-time PCR]**	
**GAPDH-qPCR Forward**	TCGCTCTCTGCTCCTCCTGTTC
**GAPDH-qPCR Reverse**	CGCCCAATACGACCAAATCC
**ORF16-qPCR Forward**	ACCAGCTTGGGTTGAGCATG
**ORF16-qPCR Reverse**	GGCTCGCCCCCAGTTC
**ORF59-qPCR Forward**	GCCCACATCCACCGACTTC
**ORF59-qPCR Reverse**	AGCCAGAAACCAAACCCGTT
**ORFK8.1-qPCR Forward**	ACAGATTCGCACAGAAATCCCT
**ORFK8.1-qPCR Reverse**	CGAACGATACGTGGGACAATTG
**EGFR-qPCR Forward**	AACACCCTGTGGAAGTACG
**EGFR-qPCR Reverse**	TCGTTGGACAGCCTTCAAGACC

*^a^* Underlined uppercase indicates mutagenesis site, and uppercase without underline indicates pEP-KanS sequence. *^b^* Underlined uppercase indicates mutagenesis site.

## Data Availability

DNA sequence data are available from the corresponding author upon reasonable request. Other data are presented in the manuscript.

## Supplementary Materials

The supporting information can be downloaded at: https://www.mdpi.com/article/10.3390/ijms24021238/s1.
