# Supplementary material for: Kaposi’s Sarcoma-Associated Herpesvirus ORF21 Enhances the Phosphorylation of MEK and the Infectivity of Progeny Virus
Source: Int J Mol Sci. 2023 Jan 8;24(2):1238. doi: 10.3390/ijms24021238 (PMC9867424; doi:10.3390/ijms24021238)

Supplementary Figure S1

(a)

Original Data of Figure.1b

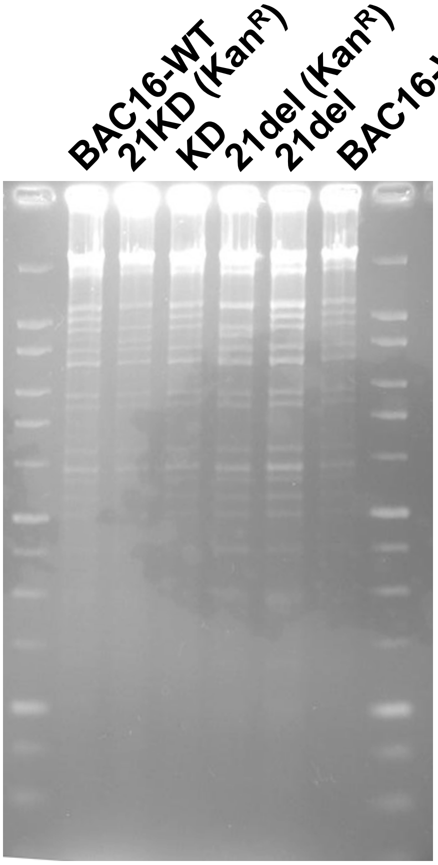

(b)

Original Data of Fig.1f

IB: ORF21

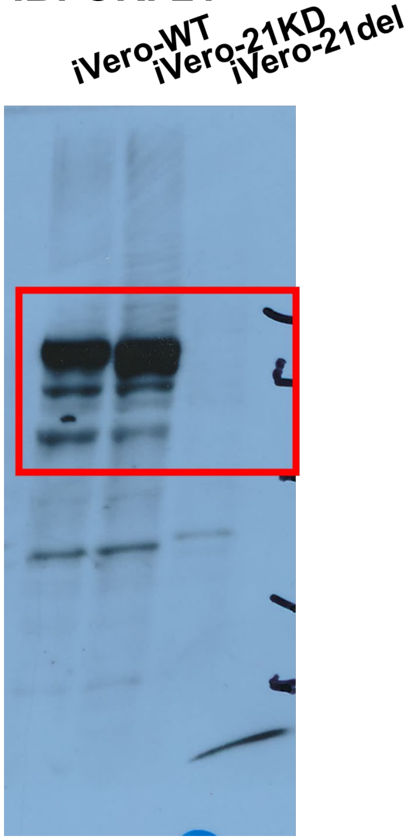

(c) Original Data of Fig.1h

IB: ORF21

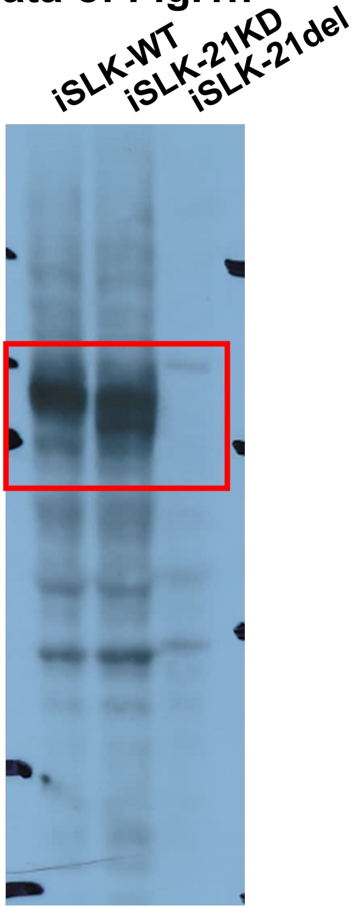

Supplementary Figure S2

Original Data of Fig.2a

IB: ORF21

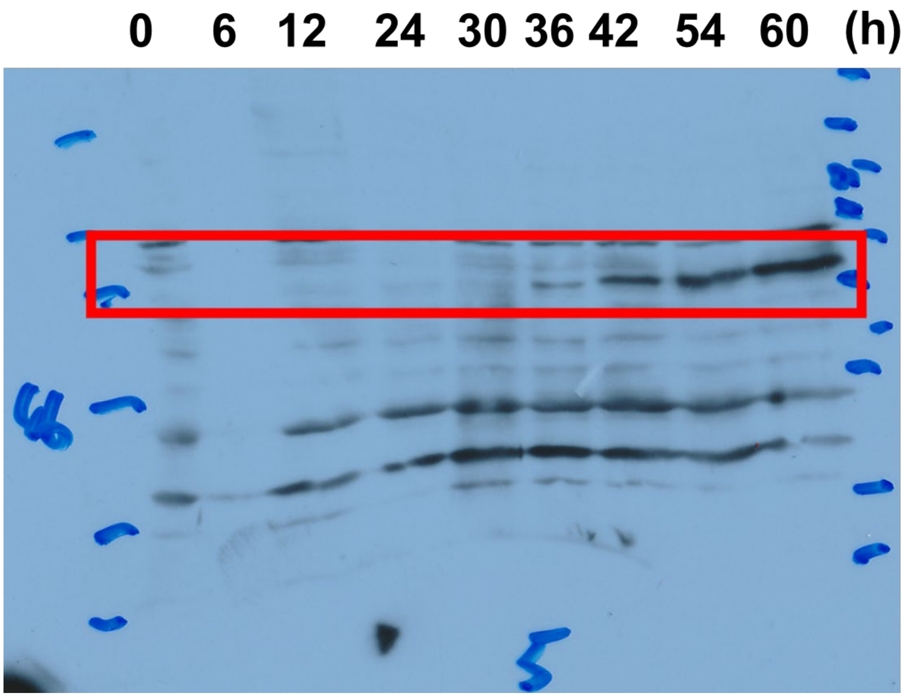

Supplementary Figure S3

Original Data of Fig.6

IB: ORF21

iSLK-WT | **iSLK-21del**

Plasmid Empty | p3F-21WT | Empty

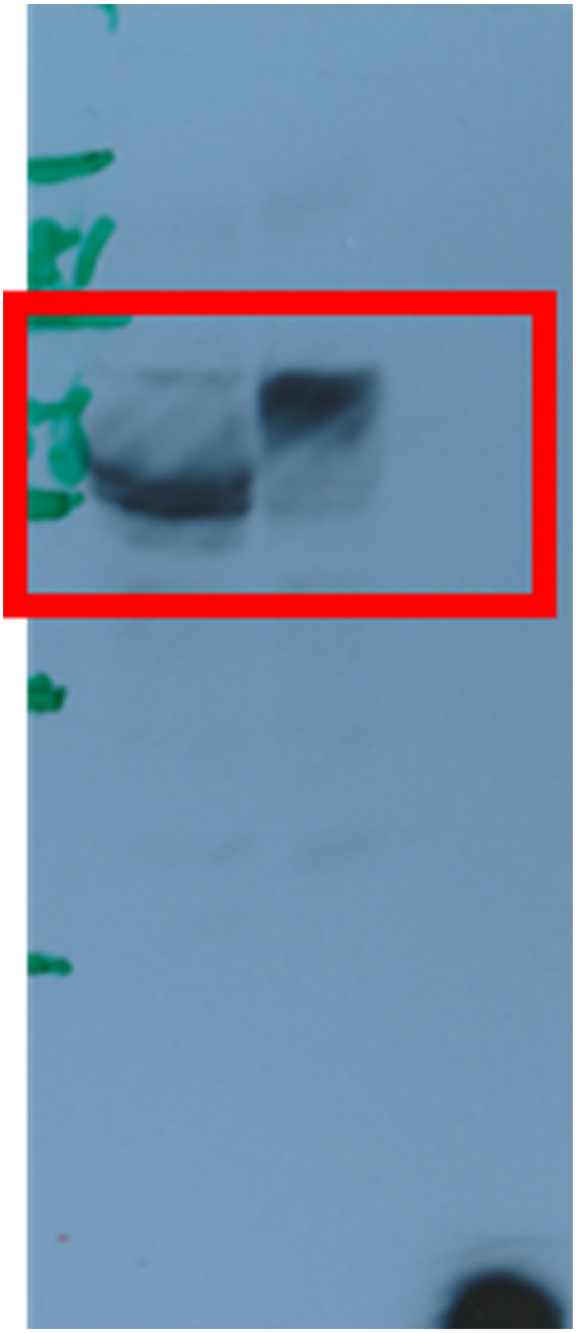

Supplementary Figure S4

(a) Original Data of Fig.7a

IB: pMEK1/2

Plasmid: Empty p3F-21WT p3F-21KD

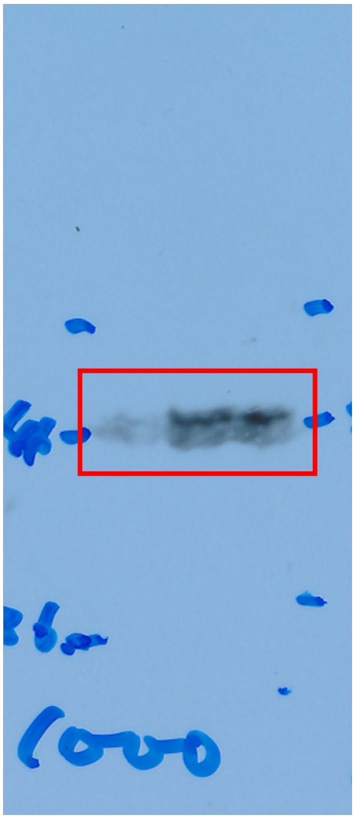

IB: pErK1/2

Plasmid: Empty p3F-21WT p3F-21KD

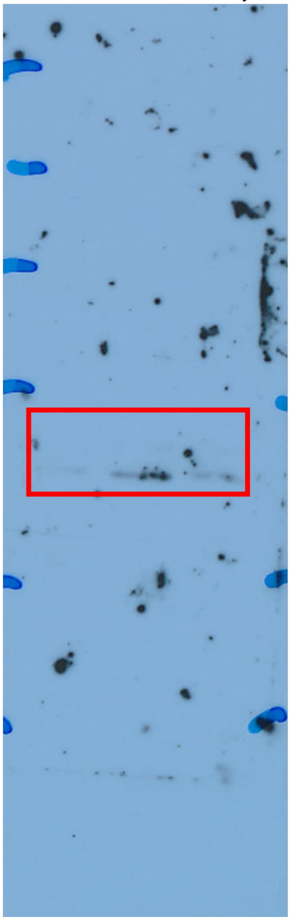

(b) Original data of Fig.7d

IB: EGFR

Dox/NaB	-				+			
iSLK-	-	WT	21KD	21del	-	WT	21KD	21del

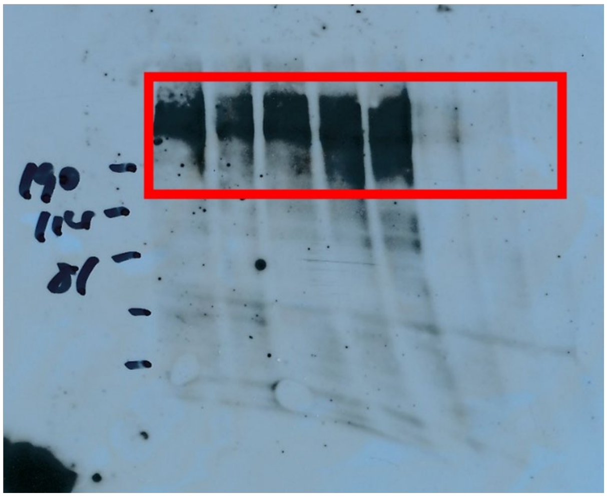

IB: pMEK1/2

Dox/NaB	-				+			
iSLK-	-	WT	21KD	21del	-	WT	21KD	21del

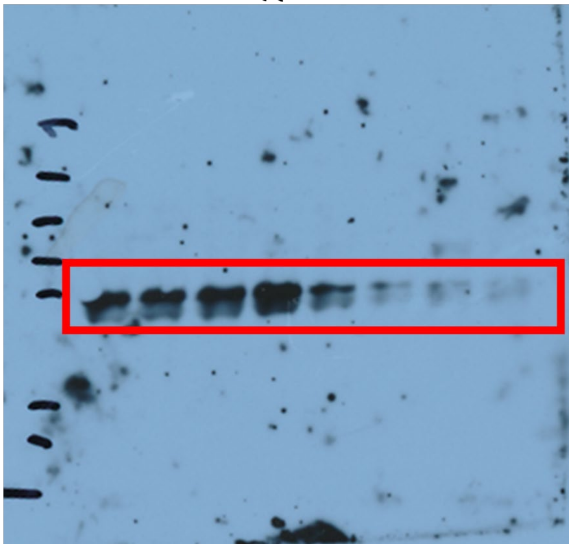

Supplement: Supplementary file 1 [file ijms-24-01238-s001.zip › ijms-2086947-supplementary.pdf]
